# Sex differences in response to violence: Role of salience network expansion and connectivity on depression

**DOI:** 10.21203/rs.3.rs-5822551/v1

**Published:** 2025-03-12

**Authors:** Ellyn R. Butler, Noelle Samia, Mandy Mejia, Damon Pham, Adam Pines, Robin Nusslock

**Affiliations:** 1 Department of Psychology, Northwestern University, Evanston, IL 60208; 2 Department of Statistics & Data Science, Northwestern University, Evanston, IL 60208; 3 Department of Statistics, Indiana University, Bloomington, IN 27205; 4 Department of Psychiatry and Behavioral Sciences, Stanford University, Palo Alto, CA 94304; 5 Institute for Policy Research, Northwestern University, Evanston, IL 60208

## Abstract

Violence is a major risk factor for depression across development. Depression quickly worsens during early adolescence, however, and especially among females, who experience worse depression following threats than males. This may be because they perceive future threats as less controllable. Evidence suggests that features of the salience network may serve as particularly critical mechanisms explaining sex differences on depression in response to threat, as those with depressive disorders have more expansive salience networks than controls, and threatening experiences result in the brain utilizing more tissue for fear generation in rodent models. Using a longitudinal sample of 220 adolescents ages 14–18 from the Chicago area, we test if salience network expansion and connectivity explain the differential impact of violence on depression across the sexes. We found that the association between violence and depression was greater for females than males $\left({\overset{ˆ}{\beta }}_{3}^{\left(2\right)}=0.337,p=0.025\right)$, such that there was a positive association among females, but not males. Contrary to our hypotheses, we found that the association between the expansion of the salience network and depression was positive for males $\left({\overset{ˆ}{\beta }}_{1}^{\left(5\right)}=0.242,p=0.039\right)$, as was the association between salience network connectivity and depression $\left({\overset{ˆ}{\beta }}_{1}^{\left(6\right)}=0.238,p=0.030\right)$. Both of these effects remained after controlling for depression two years prior, indicating that exposures that impact males’ depression through the salience network likely occur during middle adolescence. Through identifying types of exposures, their relevant developmental timing, and mechanisms connecting exposures with depression, this work helps to inform interventions to prevent the onset of depression following adversity, thereby reducing the lifetime burden of depression.

## Introduction

Violence exposure is a major risk factor for a variety of dimensions of psychopathology across development (1–5). It has been estimated that childhood adversities such as physical abuse and family violence explain nearly 30% of all psychiatric disorders during adolescence (2). This is especially worrisome because adolescence may be a sensitive period for social brain development (6), and is a time where youth are more likely to engage in maladaptive emotion regulation strategies such as rumination and suppression (7), which may be a factor in the spike in depression seen during this phase of development (8). Notably, the prevalence of violence and the effects of violence on psychopathology seem to differ across the sexes. For instance, males are more likely than females to be victims of and witness violent acts (9–16), which contrasts with the fact that females start to experience higher levels of depression than males during adolescence (8,17). Further, females reach more severe levels of internalizing psychopathology with fewer exposures to violent events (1,13,18). This apparent contradiction has led to a variety of theories to try to explain why females may suffer from more severe depression than males.

One prevalent framework for why females experience worse depression than males – the stress reactivity model – states that females are more negatively impacted by interpersonal stressors such as violence (19–22). In line with this perspective, researchers found that females who had been exposed to interpersonal violence were more likely to go on to develop Post-Traumatic Stress Disorder than males (14). Further, researchers found that both skin conductance and activity in areas of the brain responsible for threat processing responded more to threatening stimuli among women who had experienced more violence, as opposed to women who had been exposed to less violence (23). There was no evidence that previous violence exposure modulated males’ responses to threatening stimuli. Notably, females also experience more severe internalizing psychopathology than males following other types of threatening events, such as motor vehicle accidents (24,25). The stress reactivity model does not posit why females experience more severe depression than males following threatening experiences specifically, however, calling for a more tailored theory.

Females may experience worse depression following threatening events than males because they perceive future threatening events as less controllable (26). Learned helplessness research indicates that both animals and humans display greater depressive phenotypes when they do not have control over their environment (27,28). Further, researchers have demonstrated that perceived lack of control over stressors is a specific risk factor for depression (29), and that rumination mediates the association between exposure to stressors deemed uncontrollable by researchers and hopelessness among urban adolescent girls, but not their male peers (30). This suggests that adolescent girls may be more likely to perceive stressors as uncontrollable, putting them at particular risk for depression. While animal research on sex differences in response to threat is sparse, findings suggest that adolescence may be a period of development where the sexes begin to display differential susceptibility to threatening events (31). Research has shown that male adolescent rats have trouble retaining extinction learning compared to preadolescent and adult rats (32), adolescent female mice exhibit delayed extinction relative to adolescent males (33), and this trouble retaining extinction learning, in addition to freezing behavior, gets worse from adolescence to adulthood among female mice (34). Decreased extinction may ultimately lead to greater vigilance for behaviorally relevant stimuli that indicate potential threat in the environment, a function controlled in part by the salience network of the brain (35,36).

The salience network has been shown to be important for reorienting to unexpected stimuli that are behaviorally relevant (36), and as such, is often investigated in the context of threat exposure and fear learning paradigms (37–39). While many studies have shown that there are relationships between features of the salience network and threat exposure, sex, and depression, none to date have used these metrics to help understand possible neurobiological underpinnings of sex differences in response to threat. The literature suggests that exposure to threat may alter salience network properties to allow people to more efficiently detect and respond to potential threats in the environment. To this end, threat exposure has been shown to be associated with greater within salience network connectivity (40), which is thought to index greater communication within the salience network (41,42). Notably, greater communication within a network is associated with better performance on a task that utilizes that network (43), implying that threat exposure should increase connectivity within the salience network. A variety of experiences may also result in individuals utilizing more cortical tissue for a task relevant to a given experience. In line with this perspective, research has shown that the overall size of primary visual areas positively correlates with contrast sensitivity (44), that motor sequence learning leads to long-term expansion of cortical area in primary motor cortex involved in executing the sequence (45), and that a larger portion of the caudal shell of the nucleus accumbens is devoted to fear generation in threatening environments in mice (46,47). These findings suggest that experiences, including living in a stressful environment, can expand the amount of cortical tissue devoted to accomplishing tasks congruent with these experiences. Therefore, threat exposure may be associated with an expanded salience network, as experiencing threat teaches us to more efficiently detect and respond to future threats.

Alterations in the salience network may, in turn, lead to individuals experiencing more severe depression. Increases in within salience network connectivity have been found to mediate the association between abuse and higher depressive symptoms during adolescence (48), and females display more within-network connectivity than males (49). Notably, the salience network, and other association networks, have been found to take up a much larger portion of the cortical mantle – to be more expansive – among females than males (50). Further, the salience network covers twice as much of the cortical mantle among those who are depressed, compared to those who are not (51). Therefore, salience network expansion may be a particularly fruitful mechanism to examine in the pursuit to understand the neurobiological underpinnings of sex differences in response to threat.

These results converge to suggest that various features of the salience network, including connectivity and expansion, may help explain sex differences in response to threat leading to depression. To address our research questions, we studied a sample of 220 adolescents ages 14 to 18 who are from the Chicago metropolitan area. The adolescents participated in two waves spaced two years apart assessing exposure to violence, resting state functional neuroimaging, and depression. Using this sample, we tested the following hypotheses in the current work: Hypothesis #1 – Males experience more instances of violence exposure in the past year than females; Hypothesis #2 – depression increases as adolescents are exposed to more violent events, and these associations are greater among females than males; Hypothesis #3 – salience network expansion and connectivity increase as adolescents are exposed to more violent events (these associations are greater among females than males); Hypothesis #4 – depression increases as salience network expansion and connectivity increase, and these associations are greater among females than males; and Hypothesis #5 – salience network expansion and connectivity mediate the association between violent events and depression such that being exposed to more violent events is associated with greater salience network expansion and connectivity, and greater salience network expansion and connectivity are associated with more severe depression, and these effects are larger for females than males. Sensitivity analyses controlling for a variety of demographic factors are conducted. Finally, we test if the effects found in the time 2 data are robust to salience network properties and depression at time 1, which occurred two years before time 2, indicating a potential causal role of recent violent events on depression (Hyp #2) and salience network properties (Hyp #3), and recent salience network properties on depression (Hyp #4). We controlled for salience network properties and depression at time 1 because controlling for the outcome variable measured prior to the exposure of interest has been shown to reduce confounding effectively, which is essential for valid causal inference (52–54).

## Materials and Methods

### Participants

For the current project, we used the My World My Heart dataset (55–62). This dataset included violence and mental illness measures, in addition to a broad array of biological metrics that are not the focus of this paper. Recruitment prioritized adolescents from low-income neighborhoods. Between January 2015 and June 2019, 277 adolescents were recruited from the Chicago area to participate in the study. To be eligible, participants were required to be in eighth grade (typically 13–14 years old) at baseline, English-speaking, and in good health, defined as being a) not pregnant, b) without a previously diagnosed Axis I disorder, c) free of prescription medications for the past month, d) without acute infectious disease for two weeks, and e) without functional magnetic resonance imaging (fMRI) scanning contra-indications. Each child gave written assent to participate, and a parent or guardian gave written consent. Adolescents were brought back two years later for a second visit (time 2). Subjects are further excluded from analysis if they do not have 1) resting state fMRI, 2) violence exposure, or 3) depression data at the second visit. Both study sessions collected identical data, including violence exposure, neuroimaging, and depression data. Sex and race data were recorded at time 1, and body mass index percentile and income to poverty ratio were collected at both study sessions. Northwestern University’s Institutional Review Board approved the protocol.

257 adolescents returned for time 2. 29 of these subjects did not participate the neuroimaging session; 1 subject’s data failed the structural preprocessing pipeline; 6 subjects had vertices that had very low variance, rendering their estimates of salience network properties invalid; and 1 subject did not report the number of times they were exposed to violence in the past year (this subject also did not participate in the neuroimaging session). This resulted in a final sample of 220 subjects who had complete data at time 2. Of these 220 subjects, 17 did not have complete data from the first session — 3 of these subjects did not participate in the neuroimaging session; 13 subjects do not have any resting state data; and 1 subject did not obtain enough resting state data for nuisance regression. Salience network metrics missing from the 22 subjects were imputed using multiple regression models utilizing only data from time 1. Subjects who had complete data at time 2 did not significantly differ from subjects who did not have complete data at time 2 on most baseline characteristics (Black, Hispanic, Body Mass Index Percentile, Puberty Category, Income-to-Poverty Ratio, Sex, or Age). They did differ on whether or not they identified as White, however, such that among those who do not have complete data at time 2, 77% were non-White, while among those who do have complete data at time 2, 56% are non-White. Subjects detailed in Table 1 are those who had complete data at time 2 and were used for all analyses.

### Assessment

#### Violence

Violence exposure was quantified as the number of exposures to interpersonal violence that the adolescent had experienced in the past year at time 2 (63). First, adolescents were asked the following questions to assess violence exposure: 1) Have any of your family members been hurt or killed by a violent act?; 2) Have any of your friends been hurt or killed by a violent act?; 3) Have you ever seen or been present when someone was attacked with a knife or other sharp object?; 4) Have you ever seen or been present when someone was shot?; 5) Have you ever been shoved, kicked, or punched during an angry argument?; 6) Have you ever been attacked with a knife or other sharp object?; and 7) Have you ever been shot at?. If they endorsed having experienced one of these forms of violence, they were asked how many times they were exposed to that form of violence in the past year. The final measure of violence exposure is the sum of the number of exposures for each type of violence in the past year. Notably, adolescents reported on their own exposure to violence, which is important because previous studies have shown poor agreement between caretakers and adolescents on adolescents’ exposure to violence – with kappas ranging from −0.04 to 0.39 – such that caretakers consistently under-report violence exposure (63).

#### Psychopathology

Symptoms of depression and anxiety were assessed using a 25 item version of the Revised Child Anxiety and Depression Scale (64). Adolescents were asked to rate how often each item applies to them on a scale from 0 to 3 corresponding to “never”, “sometimes”, “often” and “always”. 11 items tapped into symptoms of depression. Some examples include: “I feel sad or empty”, “Nothing is much fun anymore”, and “I feel worthless”. No time frame was specified over which the adolescents should be considering their answers to the questions. The final depression measure is a sum of these 11 items.

### Neuroimaging

#### Acquisition

Imaging data were collected at Northwestern’s Center for Translational Imaging on a Siemens Prisma 3T scanner with a 64-channel phased-array head coil. Structural imaging consisted of a high-resolution navigated multiecho magnetization prepared rapid acquisition gradient echo sequence (TR = 2300ms, TEs = 1.86ms, 3.78ms; flip angle = 7°, voxel size = 0.8mm^3^). The resting state scan (T2* echoplanar imaging) utilized a fast repetition time sequence (TR = 555ms; TE = 22ms; flip angle = 47^∘^; voxel size = 2.0mm^3^; multiband factor = 8; partial Fourier Factor = 6/8; 1110 volumes).

#### Processing

Magnetic resonance imaging data were processed using fMRIPrep version 23.2.1 (65), Connectome Workbench (66), and R packages (67,68). T1-weighted images were corrected for intensity non-uniformity, and skull-stripped. Functional images were slice-timed corrected, resampled into their native space, and then projected into the fsLR-32k space (down sampled to 10k per hemispheres for computational purposes) for surface analysis. The pre-processed resting state then underwent simultaneous nuisance regression (translation (x, y, z), rotation (yaw, pitch, roll), and global signal; derivatives, second powers, and second powers of the derivatives of translation, rotation, and global signal; outlier flagging with DVARS (dual cutoff method) (69); and .01 Hz HPF using 12 discrete cosine transform bases) (70). Signal from white matter and cerebral spinal fluid were excluded because they were highly correlated with global signal across participants.

#### Network metrics

**Figure 1:**
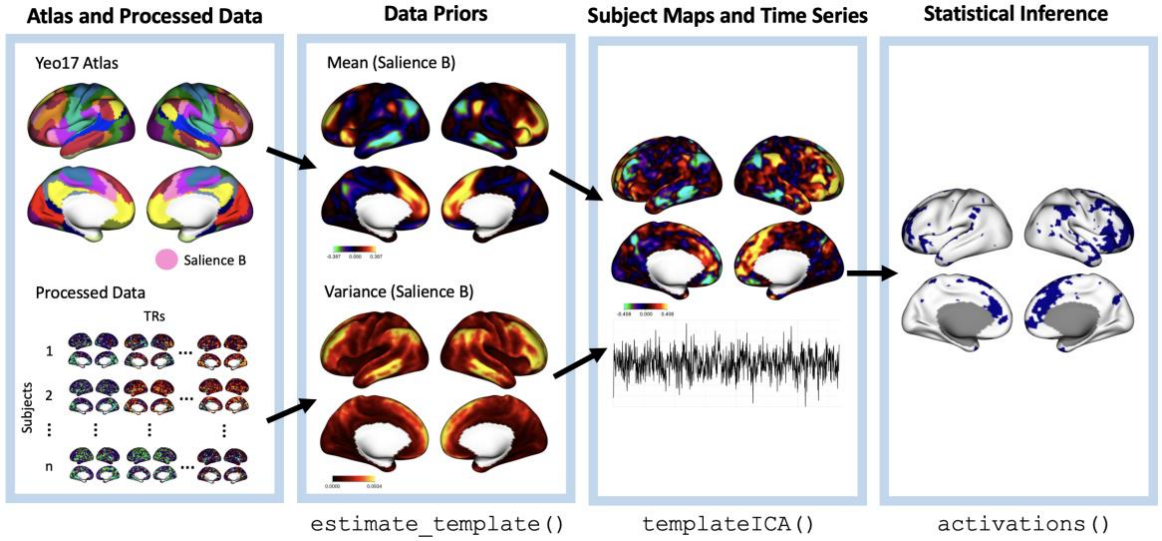
Pipeline for estimating personalized networks. Functions that produce the surface outputs in the blue boxes are listed below their corresponding boxes.

To construct individual-level network engagement maps, a Bayesian brain mapping was used (71) (Figure 1). This method employs a hierarchical Bayesian framework with population-derived priors, which can be fit for each individual separately for efficient computation. The population-derived priors result in reliable maps of individual vertex-level network engagement and membership. In this work, we used the Yeo17 atlas (72) instead of group independent component analysis (ICA) maps as in previous work (71), since we are interested in the salience network specifically, which is clearly represented in the Yeo17 atlas. Matching individual network maps to canonical group networks commonly discussed in the broader neuroimaging literature allows for sensible comparisons to be drawn. The Yeo17 atlas was used to guide the prior such that it was assumed that there would be 17 networks, and that they should resemble the topographical layout of the Yeo17 atlas. The salience network referred to in the current work corresponds to the Salience/Ventral Attention Network B in the Yeo17 atlas.

The prior mean and variance maps were derived from the 220 participants who had usable data from the second time point. Among the participants that also had resting state data available from the first time point, one session was selected at random for prior estimation, resulting in 110 sessions from the first time point and 110 sessions from the second time point being used for the prior. Each session was split down the middle to produce pseudo test-retest data, and for each pseudo-session a map of network engagement for each of the 17 networks was produced using a technique similar to dual regression (73). While dual regression is typically associated with group ICA, here we used parcels as the starting point. In the first “regression” we computed the mean time course across the vertices within each parcel, while in the second regression we regression those time courses against the fMRI time series to obtain maps of network engagement. Using the resulting set of test-retest network engagement maps for 220 subjects, the prior mean and between-subject variance maps were estimated as described previously (71). These were then used as a prior in the Bayesian model for individual network maps.

After fitting the model, we identified vertices belonging to the salience network by performing a hypothesis test at every vertex based on the estimated salience network engagement level and its standard error. We set the significance level to α=.01 and applied Bonferroni correction across all 20484 vertices. Vertices with negative engagements were not included. After identifying vertices with significant salience network engagement, we computed two summary measures: expansion and connectivity. Salience network expansion was computed as the proportion of all vertices determined to be significant members of the salience network, while salience network connectivity was computed as the average correlation in the BOLD time series between all pairs of vertices in the salience network.

#### Potential confounders

Potential confounders prior to violence exposure in the past year (pre-treatment) in the sensitivity analyses are age, sex, race, and body mass index percentile, and income to poverty ratio (74). As such, the dependent variables were all from time 2, as were the exposure variables of interest, while the control variables were from time 1, which occurred two years prior to time 2. Sensitivity analyses with depression or salience network properties at time 1 included as covariates were also conducted to determine if recent violence exposure or salience network properties have a unique effect on depression or salience network properties above and beyond these metrics two years prior. Race was quantified as self-reported racial/ethnic categories, with indicators for identifying as Black, White, or Hispanic included as covariates.

#### Statistics

A series of linear fixed-effects models were fit for n IID observations, with error terms $\epsilon_{i}^{\left(k\right)}\sim N\left(0,\sigma_{k}^{2}\right),i=1,\ldots ,n,k=1,\ldots ,8:$

$$\begin{array}{l} X_{2i}\;=\beta_{0}^{\left(1\right)}+\beta_{1}^{\left(1\right)}X_{1i}+\epsilon_{i}^{\left(1\right)},\\ Y_{i}\;=\beta_{0}^{\left(2\right)}+\beta_{1}^{\left(2\right)}X_{1i}+\beta_{2}^{\left(2\right)}X_{2i}+\beta_{3}^{\left(2\right)}X_{1i}X_{2i}+\epsilon_{i}^{\left(2\right)},\\ M_{1i}\;=\beta_{01}^{\left(3\right)}+\beta_{1}^{\left(3\right)}X_{1i}+\beta_{2}^{\left(3\right)}X_{2i}+\beta_{3}^{\left(3\right)}X_{1i}X_{2i}+\epsilon_{i}^{\left(3\right)},\\ M_{2i}\;=\beta_{0}^{\left(4\right)}+\beta_{1}^{\left(4\right)}X_{1i}+\beta_{2}^{\left(4\right)}X_{2i}+\beta_{3}^{\left(4\right)}X_{1i}X_{2i}+\epsilon_{i}^{\left(4\right)},\\ Y_{i}\;=\beta_{0}^{\left(5\right)}+\beta_{1}^{\left(5\right)}X_{1i}+\beta_{4}^{\left(5\right)}M_{1i}+\beta_{5}^{\left(5\right)}X_{1i}M_{1i}+\epsilon_{i}^{\left(5\right)},\\ Y_{i}\;=\beta_{0}^{\left(6\right)}+\beta_{1}^{\left(6\right)}X_{1i}+\beta_{4}^{\left(6\right)}M_{2i}+\beta_{5}^{\left(6\right)}X_{1i}M_{2i}+\epsilon_{i}^{\left(6\right)},\\ Y_{i}\;=\beta_{0}^{\left(7\right)}+\beta_{1}^{\left(7\right)}X_{1i}+\beta_{2}^{\left(7\right)}X_{2i}+\beta_{4}^{\left(7\right)}M_{1i}+\beta_{5}^{\left(7\right)}X_{1i}M_{1i}+\epsilon_{i}^{\left(7\right)},\\ Y_{i}\;=\beta_{0}^{\left(8\right)}+\beta_{1}^{\left(8\right)}X_{1i}+\beta_{2}^{\left(8\right)}X_{2i}+\beta_{4}^{\left(8\right)}M_{2i}+\beta_{5}^{\left(8\right)}X_{1i}M_{2i}+\epsilon_{i}^{\left(8\right)}, \end{array}$$

where $Y_{i}$ is depression severity for the i th subject, $i=1,\ldots ,n;X_{1i}$ is 1 if the subject is female and 0 if the subject is male; $X_{2i}$ is the number of violence exposures in the past year; and $M_{ji}$ is the salience network metric, j=1(expansion), 2(connectivity). A series of sensitivity analyses were conducted for all models above except for the first such that 1) age at the MRI visit (time 2), race (Black, White), Hispanic identity, body mass index percentile (time 2), puberty category (time 2), and income-to-poverty ratio (time 1) were controlled for; 2) the value of the dependent variable at time 1 was added as a control variable; and 3) the control variables from (1) and (2) were combined. The above models test the following hypotheses: (1) Hyp #1; (2) Hyp #2; (3) Hyp #3a - expansion; (4) Hyp #3b - connectivity; (5) Hyp #4a; (6) Hyp #4b; (7) Hyp #5a; and (8) Hyp #5b.

## Results

**Figure 2:**
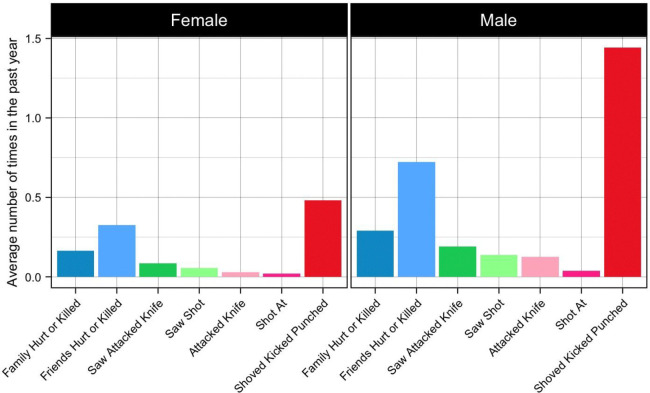
Average number of times females and males experienced each type of violence in the past year at the second time point.

**Figure 3:**
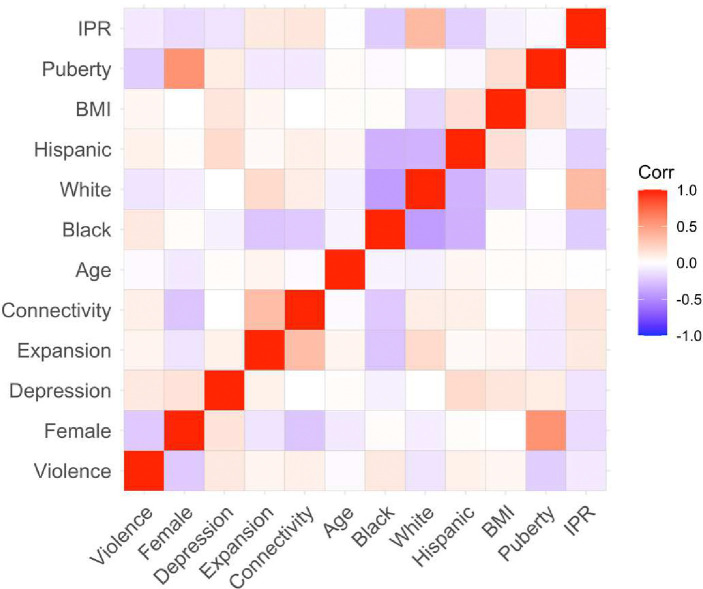
Correlations between violence, sex, depression, salience network expansion and connectivity, and demographic variables at the second time point. IPR = income-to-poverty ratio; BMI = Body Mass Index.

In the current study, we investigated a series of hypotheses pertaining to sex differences in response to violence, and potential brain mechanisms leading to depression. We addressed potential confounding by adjusting for various combinations of covariates: 1) the dependent variable time 1, which occurred two years before time 2; 2) demographic variables; and 3) the covariates from the first two sets of sensitivity analyses combined. 29% of the sample had been exposed to violence in the past year at time 2. The most common type of violence exposure was being shoved, kicked or punched during an angry argument (Figure 2). Generally, a similar pattern was seen across females and males in terms of the frequency of different exposures relative to each other, but the exposures were generally more common among males than females. The average score on the depression measure was 8.8, with values ranging from 0 to 28. Note that, the maximum possible value is 33, which would indicate that the participant constantly experiences all of the enumerated symptoms. Correlations between variables of interest and demographic variables at the second time point can be found in Figure 3.

**Figure 4:**
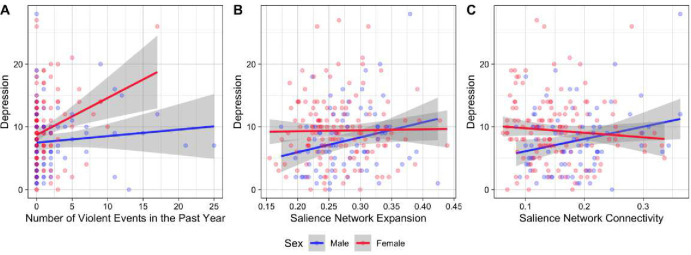
Sex interactions with A) violence, B) salience network expansion, and C) salience network connectivity leading to depression at the second time point.

On average, males were exposed to violence 2.5 times as many times as females in the past year at time 2 (females = 1.16, males = 2.95 exposures; F(1,218)=12.67,p=0.0005). Consistent with our hypotheses, females experienced more severe depression on average than males $\left({\overset{ˆ}{\beta }}_{1}^{\left(2\right)}=0.399,t=2.837,p=0.005\right)$, and the association between the number of violence exposures in the past year and depression was greater for females than males (${\overset{ˆ}{\beta }}_{3}^{\left(2\right)}=0.337,t=2.251,p=0.025$; Figure 4A), such that there was a positive association among females (r=0.264,t(139)=3.226,p=0.002), but not males (r=0.102,t(77)=0.904,p=0.369). When controlling for depression at time 1, females experienced more severe depression on average than males (${\overset{ˆ}{\beta }}_{1}^{(2)\prime }=0.251,t=2.125,p=0.035$; the single apostrophe indicates that this is a coefficient from the sensitivity analysis only controlling for the outcome variable at baseline). When controlling for demographic variables, females experienced more severe depression on average than males (${\overset{ˆ}{\beta }}_{1}^{(2)\prime \prime }=0.361,t=2.137,p=0.034$; the double apostrophe indicates that this is a coefficient from the sensitivity analysis only controlling for demographic variables), and the association between the number of violence exposures in the past year and depression was greater among females than males $\left({\overset{ˆ}{\beta }}_{3}^{(2)\prime \prime }=0.382,t=2.535,p=0.012\right)$. When controlling for depression at time 1 and demographic variables, no associations remained. For all parameter estimates, see Supplementary Table 1.

**Figure 5:**
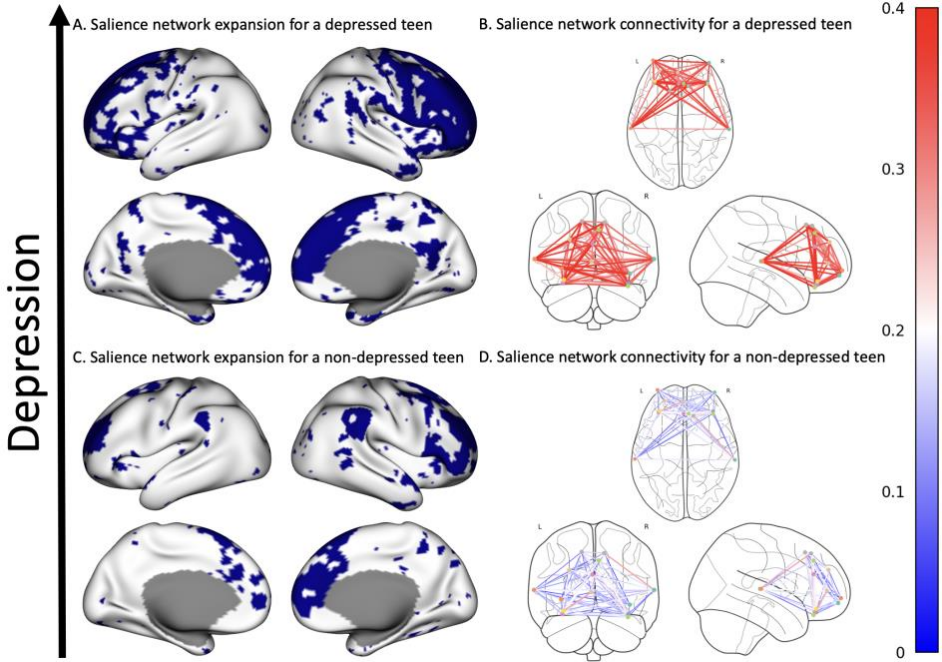
Illustration of the association between salience network metrics and depression at the second time point.

The number of violence exposures in the past year, sex, and the interaction between the two were not associated with the expansion of the salience network (Supplementary Table 2), and females have lesser connectivity within the salience network than males (Supplementary Table 3). Contrary to our hypothesis, the association between the expansion of the salience network and depression was positive for males (${\overset{ˆ}{\beta }}_{1}^{\left(5\right)}=0.242,t=2.075,\;p=0.039$; Figure 4B). This effect remained after controlling for depression at time 1, and after controlling for demographic variables, but not after controlling for both (Supplementary Table 4). Contrary to our hypothesis, the association between salience network connectivity and depression was greater among males than females (${\overset{ˆ}{\beta }}_{1}^{\left(6\right)}=0.238,\;t=2.182,p=0.030;\beta_{5}^{\left(6\right)}=-0.326,t=-2.322,p=0.0212$; Figure 4C) such that there was a positive association among males (r=0.251,t(77)=2.271,p=0.026), but not females (r=-0.082,t(139)=-0.973,p=0.332). When controlling for depression at time 1 this effect remained. The effect was not present after controlling for only demographic variables or demographic variables and depression at time 1, however. For these parameter estimates, see Supplementary Table 5. Mediation analyses were not conducted due to inconsistent exposure and mechanistic effects — violence was associated with depression among females, but salience network metrics were not associated with depression among females, and violence was not associated with depression among males, but salience network metrics were associated with depression among males. All variables that are not indicators are scaled to have mean 0, variance 1. Figure 5 illustrates the association between salience network metrics and depression among males at time 2.

## Discussion

In the current study, we proposed a model whereby threatening experiences increase salience network expansion and connectivity, which in turns worsens depression, with these effects being more pronounced among females than males. As has been demonstrated previously, we found that females experience more severe depression than males on average, and are exposed to fewer instances of violence. Consistent with our hypotheses, we found that the association between the number of violent events in the past year and depression is greater among females than males, such that there is a positive association for females, and no association for males. This effect did not remain after controlling for depression two years prior, however, indicating that the relevant exposures of interest for females may occur earlier during adolescence or during childhood.

While there was no evidence for an association between violence and salience network expansion or connectivity among females or males, there is evidence that there is a positive association between salience network expansion and connectivity with depression among males. This is contrary to our predictions, as we expected any such association to be present for females and not males. Notably, these effects were robust to some key potential confounders. Salience network expansion was positively associated with depression for males after controlling depression two years prior, and after controlling depression two years prior and demographic covariates, indicating that there is likely an exposure occurring in these teen males’ lives that increases salience network expansion and depression during this two year interval. Salience network connectivity was positively associated with depression for males after controlling for depression two years prior, suggesting that there is likely an exposure that is occurring during this two year time period that increases connectivity within the salience network and depression.

These results provide important information for determining the type and timing of adverse exposures that impact males and females to lead to depression during adolescence. For females, our results indicate that especially impactful exposures likely occur during early adolescence or childhood, considering controlling for depression two years prior renders the association between recent violence exposure and depression not significant. Violence during early adolescence or childhood may be particularly worthwhile exposure to investigate, as violence exposure is positively associated with depression for females during adolescence. For males, our results suggest that exposures that lead to depression through the salience network likely occur during a two year period, roughly between the ages of 14 and 16, considering these results remain significant when controlling for depression two years prior. Therefore, interventions to prevent exposures that lead to depression among males or to treat depression itself may be especially effective during this stage of development.

The current study is limited by measurement considerations and timing of variables with respect to one another. The violence measure is limited in that it is a count of the number of exposures to violence the adolescent has experienced in the past year. There are many important nuances to violence exposure, such as developmental timing, perpetrator, frequency, severity, controllability, and predictability that may further explain variance in depression. Therefore, we may be underestimating the magnitude of the effect that violence has on salience network metrics and depression. Future studies should collect more detailed measures of violence exposure so that the impact of these factors can be evaluated systematically. Similarly, given that the current study utilized a community sample of adolescents, depression is less severe than one would tend to find in other populations, such as help-seeking samples.

The timing of the violent events with respect to salience network metrics and depression is also largely unknown, limiting our ability to discern mechanisms. While we know that the violent events occurred in the past year, we do not know when within that time frame they occurred. The phrasing of the depression measure is also ambiguous with respect to time, as it simply asks participants how often they experience each symptom, without specifying over what time period that statement applies (75). Future research should employ frequent assessments of violence exposure and depression to help disambiguate the temporal relationships. This approach poses challenges, however, as one must both ethically and legally intervene once it has been determined that a child has been exposed to violence. If frequent assessments are not feasible, researchers should consider collecting additional data about the timing of exposures to violence, to the extent that adolescents can remember.

Despite these limitations, the current study has a number of strengths, including rigorous testing of potential confounders made possible through a longitudinal design, and use of a personalized network approach that addresses many limitations of prior methods (71). This study collected data on adolescents twice spaced two years apart, allowing us to control for depression and salience network metrics prior to exposures of interest, improving the strength of any causal claims, as the level of the outcome variable prior to the exposure of interest is typically a better control variable than demographic covariates (52–54). Considering reducing bias due to non-random assignment in observational studies is the critical issue for moving towards causal claims, this work represents a major step forward, considering many studies that investigate the impact of violence in developmental psychopathology typically do not control for psychopathology prior to violence exposure (76–79). Measurement error and lack of knowledge about the functional form of the relationship between pretreatment covariates and outcome variables nevertheless still serve as limitations to any potential causal claims (53,80,81).

Our Bayesian network mapping method represents another methodological advancement of the current study, in that it improves over a number of limitations of existing personalized network methods (71). Popular methods include dual regression (73) and group information guided (GIG)-ICA (82). As in our Bayesian method, both dual regression and GIG-ICA match the independent components across subjects and studies, and the independent components apply to unseen subjects. However, those methods have notable shortcomings in comparison. Dual regression does not reduce noise in the estimates through a prior or penalty, and as a result the engagement maps are typically noisy. GIG-ICA does reduce noise through a penalty, but it requires manually selecting a penalty value, while in a Bayesian framework the optiomal degree of shrinkage is determined automatically based on the prior variance and the properties of the data. This is not possible in GIG-ICA since the model only includes group average maps, with no measure of variance across subjects. Neither of these methods remove nuisance independent components, or perform inference via statistically principled thresholding, as our Bayesian model does. An additional advantage of our Bayesian network mapping approach is that vertices can be classified as belonging to multiple networks, unlike many existing methods that assign each vertex to belong to exactly one network (51,83,84). This represents a methodological improvement, as we know from a vast literature that there are hubs – regions that affiliate with multiple networks – in the brain (85–87).

These methodological improvements allow us to conclude with greater certainty details about salience network mechanisms connecting violence with depression across the sexes. While recent violence exposure is differentially associated with depression among males and females, after controlling for levels of depression prior to recent violence exposure, there is no association between violence and depression for either of the sexes. This indicates that if violence has a greater impact on females than males, the relevant exposures likely occur earlier in development. Therefore, if interventions to prevent violence exposure are to be effective at reducing depression among females during adolescence, they most likely need to occur earlier in development. Policymakers can use this knowledge to direct funds towards earlier interventions for violence prevention, as many existing programs focus on adolescents (88–92).

Further, salience network expansion and connectivity are positively associated with depression among males even after controlling for depression two years prior, highlighting that it is likely that males are experiencing some type of adversity that increases connectivity within the salience network, expansion of the salience network, and depression during this time period in early- to mid-adolescence. Therefore, future efforts to determine which exposures lead to depression during adolescence in males should focus during this developmental time frame. Potentially salient experiences for males may include lack of social support, bullying, and high-risk substance use (93–96). Through identifying the types of exposures, their relevant developmental timing, and the mechanisms connecting these exposures with depression, we will be better positioned to target effective interventions to prevent the onset of depression following adversity, thereby hopefully reducing the lifetime burden of depression on individuals, their communities, and society at large (97,98).

## Supplementary Material

Supplement 1

## Figures and Tables

**Table 1:** Sample characteristics by sex at the second time point

	Female (N=141)	Male (N=79)	Overall (N=220)

**Age**			
MeAn (SD)	16.0 (0.563)	16.1 (0.568)	16.1 (0.566)
MediAn [Min, Max]	16.0 [14.0, 17.5]	16.1 [15.0, 17.9]	16.1 [14.0, 17.9]
**Black**	54 (38.3%)	29 (36.7%)	83 (37.7%)
**White**	58 (41.1%)	38 (48.1%)	96 (43.6%)
**Hispanic**	42 (29.8%)	23 (29.1%)	65 (29.5%)
**BMI Percentile**			
Mean (SD)	72.1 (24.7)	71.9 (25.5)	72.0 (24.9)
Median [Min, Max]	78.5 [6.80, 99.4]	79.3 [10.4, 99.7]	78.6 [6.80, 99.7]
**Puberty Category**			
MeAn (SD)	4.43 (0.496)	3.68 (0.567)	4.16 (0.632)
MediAn [Min, Max]	4.00 [4.00, 5.00]	4.00 [2.00, 5.00]	4.00 [2.00, 5.00]
**Income to Poverty Ratio**			
MeAn (SD)	3.40 (2.79)	4.93 (7.45)	3.95 (5.03)
MediAn [Min, Max]	2.62 [0.202, 13.6]	3.20 [0, 53.0]	2.83 [0, 53.0]
**Number of Violent Events**			
MeAn (SD)	1.16 (2.37)	2.95 (5.05)	1.80 (3.66)
MediAn [Min, Max]	0 [0, 17.0]	1.00 [0, 25.0]	0 [0, 25.0]
**Depression**			
MeAn (SD)	9.38 (5.31)	7.77 (5.13)	8.80 (5.29)
MediAn [Min, Max]	9.00 [0, 27.0]	7.00 [0, 28.0]	8.00 [0, 28.0]

Note: Puberty category levels are 1 = Prepubertal, 2 = Early pubertal, 3 = Midpubertal, 4 = Late pubertal, 5 = Postpubertal; Number of violent events are in the past year.
