# Supplementary material for: Sex differences in response to violence: Role of salience network expansion and connectivity on depression
Source: Res Sq. 2025 Mar 12:rs.3.rs-5822551. Preprint. [Version 1] doi: 10.21203/rs.3.rs-5822551/v1 (PMC11952664; doi:10.21203/rs.3.rs-5822551/v1)
Supplement: Supplement 1 [file NIHPPRS5822551V1-supplement-1.pdf]

Supplementary Information

Supplementary Table 1: Effects of violence on depression by sex

	Depression			Depression			Depression			Depression		
Predictors	*Estimates*	*CI*	*p*	*Estimates*	*CI*	*p*	*Estimates*	*CI*	*p*	*Estimates*	*CI*	*p*
(Intercept)	-0.22	-0.44 – 0.00	0.053	-0.15	-0.33 – 0.04	0.113	-0.44	-0.86 – -0.02	**0.040**	-1.00	-1.74 – -0.26	**0.008**
Violence	0.07	-0.09 – 0.23	0.367	0.09	-0.04 – 0.22	0.175	0.06	-0.10 – 0.21	0.467	0.09	-0.05 – 0.22	0.210
Female	0.40	0.12 – 0.68	**0.005**	0.25	0.02 – 0.48	**0.035**	0.26	-0.07 – 0.59	0.118	0.10	-0.19 – 0.38	0.500
Violence × Female	0.34	0.04 – 0.63	**0.025**	0.11	-0.14 – 0.36	0.405	0.38	0.08 – 0.68	**0.012**	0.14	-0.12 – 0.40	0.294
Baseline Depression				0.55	0.44 – 0.66	**<0.001**				0.52	0.41 – 0.64	**<0.001**
Age							-0.02	-0.16 – 0.11	0.722	-0.03	-0.15 – 0.08	0.554
Black							0.04	-0.31 – 0.39	0.829	0.06	-0.24 – 0.36	0.708
White							0.37	0.03 – 0.72	**0.033**	0.22	-0.07 – 0.52	0.139
Hispanic							0.46	0.10 – 0.82	**0.013**	0.27	-0.04 – 0.58	0.084
BMI Percentile							0.11	-0.03 – 0.24	0.113	0.00	-0.00 – 0.01	0.796
Puberty Category							0.11	-0.05 – 0.27	0.171	0.19	-0.01 – 0.39	0.057
Income to Poverty Ratio							-0.09	-0.23 – 0.05	0.207	-0.06	-0.18 – 0.06	0.301
Observations	220			220			220			220		
R^2 / R^2 adjusted	0.070 / 0.057			0.358 / 0.346			0.146 / 0.106			0.388 / 0.355		

Supplementary Table 2: Effects of violence on salience network expansion by sex

	Expansion			Expansion			Expansion			Expansion		
Predictors	*Estimates*	*CI*	*p*	*Estimates*	*CI*	*p*	*Estimates*	*CI*	*p*	*Estimates*	*CI*	*p*
(Intercept)	0.16	-0.07 – 0.38	0.178	0.05	-0.16 – 0.26	0.643	0.17	-0.25 – 0.60	0.426	0.33	-0.50 – 1.16	0.434
Violence	-0.03	-0.19 – 0.13	0.728	-0.01	-0.16 – 0.13	0.857	-0.01	-0.17 – 0.15	0.914	0.01	-0.15 – 0.16	0.947
Female	-0.20	-0.49 – 0.08	0.159	-0.05	-0.31 – 0.22	0.722	-0.07	-0.41 – 0.27	0.680	0.03	-0.29 – 0.34	0.875
Violence × Female	0.22	-0.08 – 0.52	0.156	0.17	-0.11 – 0.45	0.235	0.30	-0.00 – 0.60	0.053	0.21	-0.08 – 0.50	0.148
Baseline Expansion				0.40	0.27 – 0.52	**<0.001**				0.36	0.23 – 0.49	**<0.001**
Age							0.03	-0.10 – 0.17	0.624	0.06	-0.07 – 0.19	0.349
Black							-0.47	-0.83 – -0.11	**0.011**	-0.39	-0.73 – -0.05	**0.024**
White							0.22	-0.13 – 0.57	0.222	0.10	-0.23 – 0.44	0.548
Hispanic							-0.04	-0.41 – 0.32	0.812	-0.16	-0.50 – 0.19	0.375
BMI Percentile							0.05	-0.09 – 0.18	0.504	0.00	-0.00 – 0.01	0.606
Puberty Category							-0.07	-0.23 – 0.10	0.424	-0.07	-0.30 – 0.15	0.527
Income to Poverty Ratio							0.02	-0.12 – 0.16	0.799	-0.03	-0.16 – 0.11	0.704
Observations	220			220			220			220		
R^2 / R^2 adjusted	0.022 / 0.009			0.175 / 0.159			0.110 / 0.067			0.219 / 0.177		

Supplementary Table 3: Effects of violence on salience network connectivity by sex

	Connectivity			Connectivity			Connectivity			Connectivity		
Predictors	*Estimates*	*CI*	*p*	*Estimates*	*CI*	*p*	*Estimates*	*CI*	*p*	*Estimates*	*CI*	*p*
(Intercept)	0.33	0.11 – 0.55	**0.004**	0.18	-0.03 – 0.38	0.092	0.63	0.21 – 1.05	**0.004**	0.64	-0.16 – 1.44	0.114
Violence	0.03	-0.13 – 0.18	0.748	-0.03	-0.17 – 0.12	0.731	0.05	-0.11 – 0.20	0.574	-0.01	-0.16 – 0.13	0.860
Female	-0.51	-0.79 – -0.23	**<0.001**	-0.29	-0.55 – -0.02	**0.032**	-0.56	-0.89 – -0.23	**0.001**	-0.29	-0.60 – 0.03	0.072
Violence × Female	0.00	-0.29 – 0.30	0.978	-0.05	-0.32 – 0.22	0.709	0.07	-0.23 – 0.37	0.646	-0.00	-0.27 – 0.27	0.996
Baseline Connectivity				0.43	0.30 – 0.55	**<0.001**				0.43	0.31 – 0.56	**<0.001**
Age							-0.07	-0.21 – 0.06	0.280	-0.06	-0.18 – 0.07	0.374
Black							-0.54	-0.89 – -0.18	**0.003**	-0.59	-0.91 – -0.26	**<0.001**
White							-0.12	-0.47 – 0.23	0.499	-0.25	-0.57 – 0.06	0.117
Hispanic							-0.03	-0.39 – 0.34	0.892	-0.07	-0.40 – 0.26	0.679
BMI Percentile							-0.04	-0.17 – 0.10	0.599	-0.00	-0.01 – 0.00	0.345
Puberty Category							0.07	-0.09 – 0.23	0.386	0.02	-0.20 – 0.23	0.885
Income to Poverty Ratio							0.07	-0.07 – 0.21	0.332	0.04	-0.09 – 0.17	0.517
Observations	220			220			220			220		
R^2 / R^2 adjusted	0.064 / 0.051			0.226 / 0.212			0.132 / 0.090			0.291 / 0.254		

Supplementary Table 4: Effects of salience network expansion on depression by sex

	Depression			Depression			Depression			Depression		
Predictors	*Estimates*	*CI*	*p*	*Estimates*	*CI*	*p*	*Estimates*	*CI*	*p*	*Estimates*	*CI*	*p*
(Intercept)	-0.23	-0.45 – -0.01	**0.041**	-0.15	-0.33 – 0.03	0.112	-0.46	-0.89 – -0.03	**0.036**	-0.93	-1.67 – -0.19	**0.014**
Expansion	0.24	0.01 – 0.47	**0.039**	0.20	0.01 – 0.39	**0.038**	0.19	-0.04 – 0.43	0.099	0.19	0.00 – 0.39	**0.048**
Female	0.34	0.07 – 0.62	**0.015**	0.22	-0.01 – 0.44	0.063	0.20	-0.13 – 0.53	0.235	0.06	-0.22 – 0.34	0.691
Expansion × Female	-0.23	-0.51 – 0.05	0.114	-0.18	-0.41 – 0.05	0.128	-0.16	-0.44 – 0.12	0.258	-0.16	-0.39 – 0.08	0.193
Baseline Depression				0.56	0.46 – 0.67	**<0.001**				0.55	0.43 – 0.66	**<0.001**
Age							0.01	-0.13 – 0.14	0.935	-0.03	-0.14 – 0.09	0.645
Black							0.13	-0.23 – 0.50	0.477	0.12	-0.18 – 0.43	0.418
White							0.30	-0.05 – 0.66	0.093	0.17	-0.13 – 0.46	0.263
Hispanic							0.47	0.10 – 0.84	**0.013**	0.27	-0.05 – 0.58	0.094
BMI Percentile							0.09	-0.04 – 0.22	0.189	-0.00	-0.00 – 0.00	0.984
Puberty Category							0.10	-0.06 – 0.27	0.206	0.19	-0.01 – 0.39	0.065
Income to Poverty Ratio							-0.10	-0.24 – 0.04	0.162	-0.07	-0.19 – 0.04	0.219
Observations	220			220			220			220		
R^2 / R^2 adjusted	0.041 / 0.027			0.356 / 0.344			0.108 / 0.065			0.383 / 0.351		

Supplementary Table 5: Effects of salience network connectivity on depression by sex

	Depression			Depression			Depression			Depression		
Predictors	*Estimates*	*CI*	*p*	*Estimates*	*CI*	*p*	*Estimates*	*CI*	*p*	*Estimates*	*CI*	*p*
(Intercept)	-0.27	-0.50 – -0.05	**0.019**	-0.19	-0.38 – -0.00	**0.049**	-0.51	-0.94 – -0.07	**0.024**	-0.80	-1.55 – -0.05	**0.036**
Connectivity	0.24	0.02 – 0.45	**0.030**	0.21	0.04 – 0.39	**0.018**	0.18	-0.04 – 0.40	0.117	0.18	-0.00 – 0.36	0.054
Female	0.37	0.08 – 0.65	**0.011**	0.24	0.01 – 0.47	**0.044**	0.26	-0.09 – 0.61	0.146	0.11	-0.18 – 0.41	0.441
Connectivity × Female	-0.33	-0.60 – -0.05	**0.021**	-0.30	-0.53 – -0.07	**0.010**	-0.23	-0.51 – 0.05	0.109	-0.24	-0.48 – -0.01	**0.042**
Baseline Depression				0.57	0.46 – 0.67	**<0.001**				0.55	0.43 – 0.66	**<0.001**
Age							0.01	-0.13 – 0.15	0.889	-0.02	-0.14 – 0.09	0.698
Black							0.11	-0.26 – 0.48	0.554	0.10	-0.21 – 0.40	0.526
White							0.31	-0.04 – 0.67	0.081	0.18	-0.12 – 0.47	0.235
Hispanic							0.45	0.08 – 0.82	**0.017**	0.25	-0.06 – 0.56	0.119
BMI Percentile							0.10	-0.04 – 0.23	0.161	0.00	-0.00 – 0.00	0.948
Puberty Category							0.07	-0.09 – 0.24	0.399	0.14	-0.07 – 0.34	0.187
Income to Poverty Ratio							-0.10	-0.25 – 0.04	0.153	-0.08	-0.19 – 0.04	0.204
Observations	220			220			220			220		
R^2 / R^2 adjusted	0.047 / 0.034			0.363 / 0.351			0.108 / 0.065			0.384 / 0.352		
